# Comparative Study of Hypertension Prevalence, Health‐Related Quality of Life, and Healthcare Barriers Among Diabetic and Non‐Diabetic Populations in Urban Slums of Bangladesh: A Community‐Based Comparative Cross‐Sectional Study

**DOI:** 10.1002/hsr2.73120

**Published:** 2026-08-26

**Authors:** Md. Fakhrul Islam Maruf, Nishat Tamanna Omi, Mahfuza Mubarak

**Affiliations:** ^1^ Department of Public Health and Informatics Jahangirnagar University Savar, Dhaka Bangladesh

**Keywords:** Bangladesh, diabetes, EQ‐5D‐5L, healthcare barriers, health‐related quality of life, hypertension, urban slums

## Abstract

**Background and Aims:**

The double burden of diabetes and hypertension is a growing public health concern in Bangladesh's urban slums, where healthcare access and health‐related quality of life (HRQoL) remain limited. This study compared hypertension prevalence, HRQoL, and healthcare barriers between adults with and without self‐reported diabetes, and identified factors independently associated with hypertension and HRQoL in this population.

**Methods:**

A community‐based comparative cross‐sectional study was conducted from June to August 2025 in five purposively selected urban slums of Dhaka. Using simple random sampling, 400 adults aged ≥ 45 years (200 with self‐reported diabetes, 200 without) were recruited. Data were collected using the validated Bengali WHO STEPS questionnaire and the EQ‐5D‐5L instrument. Multivariable logistic regression identified factors independently associated with self‐reported hypertension (the primary, prespecified outcome), with diabetes status entered as the key exposure variable, and multivariable linear regression examined factors associated with the EQ‐5D‐5L index score.

**Results:**

Hypertension was more prevalent among participants with diabetes (121/200, 60.5%) than without (46/200, 23.0%; *χ* ^2^ = 57.8, *p* < 0.001). After adjustment, diabetes remained the strongest independent correlate of hypertension (adjusted odds ratio [aOR] 3.82, 95% CI 2.46–5.93, *p* < 0.001), alongside older age, higher body mass index, smoking, and non‐manual occupation. Diabetic participants had significantly lower EQ‐5D‐5L index scores (0.72 ± 0.18 vs. 0.85 ± 0.12, *p* < 0.001); this association persisted after adjustment (*β* = −0.081, 95% CI − 0.104 to −0.058, *p* < 0.001). Among participants with diabetes, financial constraints were the leading healthcare barrier (167/200, 83.5%).

**Conclusion:**

Diabetes is strongly and independently associated with hypertension and impaired HRQoL among older adults in Dhaka's urban slums. Financial constraints dominate healthcare access barriers. Equity‐focused, integrated NCD interventions targeting this vulnerable population are urgently needed.

## 1 Introduction

Non‐communicable diseases (NCDs) have emerged as the leading cause of death and disability worldwide, accounting for nearly 75% of all non‐pandemic‐related deaths globally [1, 2]. Among NCDs, diabetes mellitus and hypertension represent two of the most prevalent and clinically significant chronic conditions due to their lifelong nature, frequent coexistence, and association with severe complications such as cardiovascular disease, stroke, chronic kidney disease, neuropathy, and retinopathy [3, 4]. Recent estimates indicate that approximately 589 million adults were living with diabetes in 2024, and this number is projected to increase to 853 million by 2050 [5]. Similarly, hypertension affects more than 1.4 billion adults aged 30–79 years worldwide, with the greatest burden concentrated in low‐ and middle‐income countries (LMICs) [6, 7]. The coexistence of diabetes and hypertension substantially elevates the risk of morbidity, disability, healthcare expenditure, and premature mortality compared with either condition alone [8].

LMICs currently bear more than 73% of the global burden of NCDs, largely driven by rapid urbanization, demographic transition, sedentary lifestyles, unhealthy dietary practices, tobacco use, and limited access to preventive healthcare services [9, 10]. Bangladesh, a densely populated lower‐middle‐income country in South Asia, is experiencing a rapid epidemiological transition characterized by a declining burden of communicable diseases alongside a growing prevalence of chronic non‐communicable conditions. National findings from the 2018 STEPS survey reported the prevalence of diabetes and hypertension among Bangladeshi adults to be 9.8% and 27.3%, respectively, both demonstrating increasing trends over recent years [11, 12]. Urban populations, particularly residents of Dhaka city, are disproportionately exposed to NCD risk factors because of obesogenic environments, occupational stress, reduced physical activity, unhealthy food environments, and changing lifestyle behaviors associated with urban living [13].

Urban slum populations represent one of the most socioeconomically vulnerable and rapidly expanding groups in Bangladesh. Dhaka alone is estimated to accommodate approximately 3.4–14 million slum residents, constituting nearly 40% of the city's total urban population [14, 15]. These communities are characterized by overcrowding, extreme poverty, poor housing conditions, inadequate sanitation, environmental pollution, food insecurity, chronic psychosocial stress, and severely constrained healthcare access. Such adverse living conditions may contribute not only to a higher burden of chronic diseases but also to delayed diagnosis, inadequate disease management, and poorer health outcomes. Guided by the social determinants of health framework, which recognizes the influence of socioeconomic deprivation, environmental conditions, and healthcare accessibility on chronic disease outcomes and quality of life, understanding NCD patterns in slum settings is essential for developing equitable public health interventions.

Previous studies conducted in Bangladesh and other LMIC slum settings have reported considerable burdens of diabetes and hypertension among disadvantaged urban populations [16, 17]. Studies from Dhaka slums documented hypertension prevalence ranging from 28% to 34% and diabetes prevalence between 5% and 14% among adults, rates often exceeding those observed in rural populations [16, 17]. Existing evidence further suggests that individuals living with diabetes frequently experience coexisting hypertension, reduced health‐related quality of life (HRQoL), and greater healthcare utilization challenges [18, 19]. However, findings across studies remain inconsistent because of variations in study design, sampling strategies, measurement approaches, and population characteristics. Moreover, most previous research in Bangladesh has examined diabetes and hypertension separately rather than assessing their coexistence and comparative burden within marginalized urban communities.

Despite the growing body of literature on NCDs in Bangladesh, several important knowledge gaps remain. First, limited evidence exists comparing the prevalence of hypertension between diabetic and non‐diabetic populations residing in urban slums. Second, few studies have comprehensively examined HRQoL and healthcare access barriers in relation to diabetes status using validated instruments such as the EQ‐5D‐5L. Furthermore, the relative contribution of financial, geographic, and awareness‐related barriers to healthcare access among these vulnerable populations remains poorly understood. This lack of comprehensive comparative evidence restricts the ability of policymakers and healthcare providers to design targeted interventions for high‐risk urban communities.

In the present study, diabetes status was treated as the primary grouping (exposure) variable and hypertension as the outcome, rather than the reverse. This unidirectional approach was chosen for three reasons. First, from a pathophysiological standpoint, diabetes typically precedes or co‐develops with hypertension through shared mechanisms including insulin resistance, chronic low‐grade inflammation, endothelial dysfunction, and activation of the renin–angiotensin–aldosterone system, all of which promote vascular stiffening and elevated blood pressure; hypertension is therefore frequently conceptualized in the clinical and epidemiological literature as a downstream consequence of diabetes rather than vice versa. Second, from a sampling standpoint, individuals with diagnosed diabetes in urban Bangladesh are more readily and reliably identifiable through community diabetes registers, medication records, and diabetic association contacts, providing a more verifiable exposure classification than self‐reported hypertension, for which no equivalent community‐level registry infrastructure exists. Third, from a policy standpoint, national NCD control efforts in Bangladesh are increasingly organized around diabetes‐care platforms (e.g., diabetic association clinics), making it programmatically useful to characterize the hypertension burden and its correlates specifically within the diabetic population, so as to inform integrated screening within these existing service platforms. We acknowledge that the converse approach—examining diabetes prevalence among hypertensive versus normotensive individuals—is equally scientifically valid; it was not pursued here because of the absence of a comparable community‐based hypertension registry to serve as an exposure‐ascertainment frame, and it represents an important direction for future research.

Therefore, guided by existing epidemiological evidence on NCD comorbidity and healthcare disparities, this community‐based cross‐sectional study was conducted in urban slums of Dhaka to address these research gaps. The primary objective was to determine and compare the prevalence of self‐reported hypertension among adults with and without self‐reported diabetes, and to identify factors independently associated with hypertension. The secondary objectives were to (1) compare health‐related quality of life using the EQ‐5D‐5L instrument and identify factors independently associated with HRQoL, and (2) identify and rank major barriers to healthcare access among participants with diabetes.

## 2 Methodology

### 2.1 Study Design

This community‐based comparative cross‐sectional study was conducted to assess the prevalence of self‐reported hypertension, health‐related quality of life (HRQoL), and healthcare access barriers among adults with and without self‐reported diabetes in urban slums of Dhaka, Bangladesh. A cross‐sectional design was chosen to provide a snapshot of the current burden and associations at a single time point, which is suitable for estimating prevalence and exploring associations in resource‐limited settings. Diabetes status was used as the basis for stratified (equal‐allocation) recruitment, while hypertension—which was not sampled in a fixed proportion—was treated as the outcome for all inferential (regression) analyses, consistent with a comparative cross‐sectional design in which one condition is used to define comparison groups and a second condition is compared between them.

### 2.2 Study Setting

The study was carried out in five purposively selected urban slums in Dhaka city: Mirpur, Khilgaon, Korail, Bhashantek, and Kamalapur. These slums were selected because they represent typical socio‐economic and environmental conditions of Dhaka's informal settlements, including high population density, overcrowding, poor sanitation, environmental pollution, and limited healthcare access. Dhaka hosts an estimated 3.4–14 million slum dwellers, comprising around 40% of the city's population [BBS, 2022].

### 2.3 Participants

Participants were adults aged 45 years and above residing in the selected slums for at least 1 year. The minimum age of 45 years was selected because national STEPS and community‐screening data from Bangladesh indicate a marked acceleration in diabetes and hypertension incidence beyond this threshold, and because local diabetic association registers—used to help identify the diabetic subgroup—predominantly enroll adults in this age range, providing a feasible and verifiable sampling frame. We acknowledge that a smaller proportion of younger adults (< 45 years) also develop diabetes and hypertension in this population; restricting recruitment to adults ≥ 45 years means that our findings describe the burden and correlates of these conditions specifically among older slum residents and should not be extrapolated to younger‐onset disease, a limitation discussed further in Section Conclusion, 4. Both individuals with self‐reported diabetes (previously diagnosed by a qualified healthcare provider) and those without were included. Exclusion criteria were: severe acute illness at the time of interview, inability to provide informed consent, or severe cognitive impairment that would prevent reliable responses.

### 2.4 Sample Size and Sampling

The sample size was calculated using the single population proportion formula for prevalence estimation: *n* = *Z* ^2^ *p* (1−*p*)/*d* ^2^ where *Z* = 1.96 (95% confidence level), *p* = 0.10 (expected diabetes prevalence), and *d* = 0.05 (margin of error). This yielded approximately 139 participants per group. The sample was increased to 200 per group (total *n* = 400) to ensure adequate power for multivariable logistic regression and subgroup comparisons.

A two‐stage sampling technique was employed. In the first stage, five slums were purposively selected. In the second stage, household lists were prepared with the help of local community leaders and organizations in each slum. Eligible participants were then selected using simple random sampling within each slum using a computer‐generated random number list. An equal number of diabetic and non‐diabetic participants (200 each) were enrolled through this process; this equal allocation applies to the diabetes exposure variable only and does not constrain the distribution of hypertension, which was measured as an outcome within this sample (Section 2.1).

### 2.5 Data Collection

Trained interviewers conducted face‐to‐face interviews using a structured questionnaire. The questionnaire was adapted from the validated Bengali version of the WHO STEPwise Approach to Surveillance (STEPS) instrument for NCD risk factors. It collected information on socio‐demographic characteristics, behavioral risk factors, self‐reported medical history, and healthcare barriers.

Hypertension status was assessed by asking: “Have you ever been told by a doctor or other health professional that you have high blood pressure (hypertension)?” Blood pressure was not directly measured at the time of interview because of logistical constraints in the slum field setting, including the absence of calibrated equipment, cold‐chain requirements, certified phlebotomists, and safe referral pathways for abnormal readings; this is discussed as a limitation in Section Conclusion, 4.

Diabetes status was based on self‐reported physician diagnosis. Where available, interviewers additionally requested to see supporting documentation of the diagnosis (physician prescriptions, glucometer or laboratory reports, or diabetic association registration cards); such documentation was available and reviewed for 156 of 200 (78.0%) diabetic participants, supporting the validity of self‐report for the majority of this subgroup. For the remaining participants, diagnosis was accepted on self‐report alone, consistent with standard STEPS methodology.

**Figure 1:**
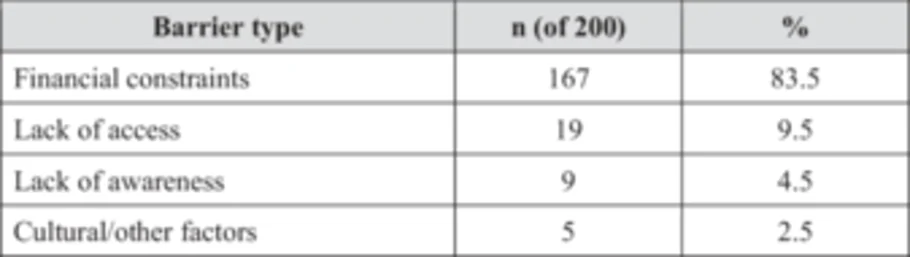
Proportion of self‐reported barriers to healthcare access among the 200 participants with self‐reported diabetes. Because ongoing healthcare contact is a near‐universal requirement for diabetes management, the barriers question was administered only within the diabetic subgroup (*n* = 200); percentages use this subgroup as the denominator (see Section 2.5).

Body mass index (BMI) was calculated from height and weight measured using standardized and calibrated instruments. Healthcare barriers were assessed using a multi‐response question and categorized into financial constraints, lack of access, lack of awareness, and cultural/other factors. Because ongoing healthcare contact for medication refills and clinical monitoring is a near‐universal requirement among individuals with diagnosed diabetes—whereas non‐diabetic participants without a hypertension diagnosis may not have required or sought care during the recall period—the healthcare‐barriers question was administered only to the 200 participants with self‐reported diabetes. This restriction, and its rationale, is also stated in the Figure 1 legend.

HRQoL was measured using the validated Bengali version of the EQ‐5D‐5L instrument, which assesses five dimensions (mobility, self‐care, usual activities, pain/discomfort, and anxiety/depression) on five levels each. The EQ‐5D index score was calculated using the recommended value set.

The questionnaire was pilot‐tested on 30 slum residents (not included in the final sample) to assess clarity, cultural suitability, and feasibility. Data were collected between June and August 2025.

### 2.6 Variables and Measurements

The primary, prespecified outcome was self‐reported hypertension (diagnosis/treatment); the secondary, prespecified outcome was HRQoL (EQ‐5D‐5L index and domain scores). Healthcare barriers (categorized responses, diabetic subgroup only) were examined descriptively. The primary exposure was diabetes status (self‐reported, documentation‐supported for 78.0% of cases); additional covariates included socio‐demographic characteristics, BMI (measured height/weight), and lifestyle factors. All variables followed WHO STEPS definitions where applicable.

### 2.7 Data Analysis

Data were analyzed using R software (version 4.4.1; R Foundation for Statistical Computing, Vienna, Austria). Descriptive statistics were presented as frequencies and percentages, reported together with their numerators and denominators, for categorical variables, and as means ± standard deviations for normally distributed continuous variables. Bivariate comparisons between diabetic and non‐diabetic groups were performed using chi‐square tests for categorical variables and independent‐samples *t*‐tests for continuous variables.

The primary, prespecified analysis was a multivariable logistic regression model with self‐reported hypertension as the outcome. Diabetes status was entered as the key exposure variable. Candidate covariates (age group, sex, BMI category, education, marital status, occupation, income group, and smoking status) were selected a priori based on established literature and were retained in the final adjusted model if they showed an association with hypertension at *p* < 0.20 in bivariate screening, following standard model‐building practice; this variable‐selection step, together with additional lifestyle covariates examined only in Table 1/bivariate analysis (dietary patterns, walking frequency, and type of physical work), was exploratory rather than prespecified for the final adjusted model. Variance inflation factors (all < 2) confirmed the absence of problematic multicollinearity. Model fit was assessed using the Hosmer–Lemeshow goodness‐of‐fit test and Nagelkerke *R* ^2^. With 167 hypertension events among 400 participants and 8 covariates (approximately 16 estimated parameters) in the final model, the resulting events‐per‐variable ratio (≈10) met conventional adequacy thresholds for multivariable logistic regression [Peduzzi et al.]. Crude and adjusted odds ratios (OR/aOR) with 95% confidence intervals are reported.

**Table 1:** Socio‐demographic characteristics of the participants, by diabetes status.

Variable	Level	Diabetic (*n* = 200)	Non‐diabetic (*n* = 200)
Sex	Male	100 (50.0)	100 (50.0)
	Female	100 (50.0)	100 (50.0)
Age group (years)	45–54	70 (35.0)	62 (31.0)
	55–64	65 (32.5)	56 (28.0)
	≥ 65	65 (32.5)	82 (41.0)
Educational qualification	No formal education	62 (31.0)	58 (29.0)
	Primary	106 (53.0)	105 (52.5)
	Secondary	29 (14.5)	35 (17.5)
	Higher secondary	3 (1.5)	2 (1.0)
	Graduate	0 (0.0)	0 (0.0)
Marital status	Married	154 (77.0)	160 (80.0)
	Unmarried	21 (10.5)	16 (8.0)
	Widowed	22 (11.0)	24 (12.0)
	Divorced	3 (1.5)	0 (0.0)
Occupation	Manual	110 (55.0)	123 (61.5)
	Non‐manual	90 (45.0)	77 (38.5)
Income status	High income	28 (14.0)	73 (36.5)
	Middle income	139 (69.5)	103 (51.5)
	Low income	33 (16.5)	24 (12.0)

A secondary, prespecified multivariable linear regression model examined factors associated with the continuous EQ‐5D‐5L index score, adjusting for diabetes status and the same covariate set described above. Model assumptions (linearity, homoscedasticity, and normality of residuals) were assessed graphically and were considered acceptable.

Site‐level (slum‐specific) hypertension prevalence was examined descriptively as an exploratory analysis; because per‐site sample sizes (*n* = 80 per slum) were not powered for formal between‐site inferential comparison, no hypothesis tests were applied to these site‐level estimates.

All statistical tests were two‐sided, with *p* < 0.05 considered statistically significant. *p*‐values are reported as *p* < 0.001 where applicable, to three decimal places for 0.001 ≤ *p* < 0.01, and to two decimal places for *p* ≥ 0.01, consistent with current statistical reporting conventions [Assel et al., 2018]. Missing data were minimal (< 5%) and handled using complete‐case analysis.

### 2.8 Ethical Considerations

The study protocol was approved by the Institutional Review Board of Jahangirnagar University (Ref: BBEC, JU/M 2025/09 (314)). Written or verbal informed consent (with impartial witness when needed) was obtained from all participants after providing detailed information in Bengali. Participation was voluntary, and participants could withdraw at any time without consequences. Confidentiality was maintained through anonymization and secure data storage. No financial incentives were provided, though participants received health education and referral information when appropriate.

## 3 Results

The study included 400 participants: 200 with self‐reported diabetes and 200 without (Table 1). The two groups were similar in sex distribution (50.0% male in each group) and in overall educational attainment, both predominantly comprising individuals with primary‐level or no formal education. Non‐diabetic participants were somewhat older on average and more frequently in the ≥ 65‐year category (41.0% vs. 32.5%), while diabetic participants were more often in the youngest, 45–54‐year stratum (35.0% vs. 31.0%). Occupational status was predominantly manual labour in both groups, though non‐manual (sedentary) occupations were more common among diabetic participants (45.0% vs. 38.5%). A notably higher proportion of non‐diabetic participants belonged to the high‐income category (36.5% vs. 14.0%), while middle‐income status predominated among diabetic participants (69.5% vs. 51.5%). Full category‐level frequencies are presented in Table 1.

Among the 200 participants with diabetes, 121 (60.5%) reported a diagnosis of hypertension, compared with 46 of 200 (23.0%) non‐diabetic participants—a statistically significant difference (*χ* ^2^ = 57.8, df = 1, *p*< 0.001; crude OR 5.13, 95% CI 3.41–7.72). This unadjusted association is examined further, with adjustment for potential confounders, in the multivariable logistic regression model presented in Table 3.

In the multivariable logistic regression model predicting self‐reported hypertension (Table 3), diabetes status was the strongest independent correlate: participants with diabetes had nearly fourfold higher adjusted odds of hypertension compared with non‐diabetic participants (aOR 3.82, 95% CI 2.46–5.93, *p* < 0.001), consistent with the crude association reported in Table 2.

**Table 2:** Comparison of self‐reported hypertension between diabetic and non‐diabetic participants.

Group	*n*	Hypertension, *n* (%)	No hypertension, *n* (%)	*χ* ^2^ (df)	*p*‐value
Diabetic	200	121 (60.5)	79 (39.5)	57.8 (1)	< 0.001
Non‐diabetic	200	46 (23.0)	154 (77.0)		

*Note:* Percentage calculated as (Hypertension n/Total n) × 100. *χ* ^2^ = Pearson *χ* ^2^ statistic.

Older age was independently associated with higher odds of hypertension: compared with the 45–54‐year group, participants aged 55–64 years had 1.68 times higher adjusted odds (95% CI 1.02–2.76, *p* = 0.04), and those aged ≥ 65 years had 2.35 times higher adjusted odds (95% CI 1.42–3.89, *p* < 0.001).

Body mass index showed a graded positive association with hypertension: overweight participants had 1.87 times higher adjusted odds (95% CI 1.19–2.94, *p* = 0.007), and obese participants had 2.62 times higher adjusted odds (95% CI 1.51–4.55, *p* < 0.001), compared with participants of normal weight.

Participants in non‐manual (sedentary) occupations had 1.58 times higher adjusted odds of hypertension (95% CI 1.02–2.45, *p* = 0.04) compared with those in manual labour, and current smokers had 1.79 times higher adjusted odds (95% CI 1.09–2.94, *p* = 0.02) compared with non‐smokers.

Sex, educational attainment, marital status, and income group were not independently associated with hypertension in the adjusted model (Table 3).

**Table 3:** Multivariable logistic regression analysis of factors associated with self‐reported hypertension.

Characteristic	Crude OR (95% CI)	*p*	Adjusted OR (95% CI)	*p*
Diabetes status				
No (Ref)	1.00	—	1.00	—
Yes	5.13 (3.41–7.72)	< 0.001	3.82 (2.46–5.93)	< 0.001
Age group				
45–54 (Ref)	1.00	—	1.00	—
55–64	1.82 (1.14–2.92)	0.01	1.68 (1.02–2.76)	0.04
≥ 65	2.61 (1.65–4.13)	< 0.001	2.35 (1.42–3.89)	< 0.001
Sex				
Male (Ref)	1.00	—	1.00	—
Female	0.96 (0.66–1.38)	0.81	0.94 (0.63–1.40)	0.75
BMI status				
Normal weight (Ref)	1.00	—	1.00	—
Overweight	2.05 (1.32–3.18)	0.001	1.87 (1.19–2.94)	0.007
Obese	2.94 (1.71–5.05)	< 0.001	2.62 (1.51–4.55)	< 0.001
Education				
No formal education (Ref)	1.00	—	1.00	—
Primary	0.90 (0.58–1.40)	0.64	0.88 (0.56–1.39)	0.59
Secondary & above	0.73 (0.44–1.22)	0.23	0.71 (0.42–1.19)	0.19
Marital status				
Married (Ref)	1.00	—	1.00	—
Others (unmarried/widowed/divorced)	1.10 (0.71–1.71)	0.67	1.06 (0.66–1.70)	0.82
Occupation				
Manual labour (Ref)	1.00	—	1.00	—
Non‐manual (sedentary)	1.71 (1.13–2.59)	0.01	1.58 (1.02–2.45)	0.04
Income group				
High income (Ref)	1.00	—	1.00	—
Middle income	1.28 (0.80–2.06)	0.31	1.22 (0.75–1.99)	0.42
Low income	1.72 (0.97–3.05)	0.06	1.61 (0.90–2.88)	0.11
Smoking status				
No (Ref)	1.00	—	1.00	—
Yes	1.94 (1.21–3.11)	0.01	1.79 (1.09–2.94)	0.02

*Note:* Model statistics: Nagelkerke *R* ^2^ = 0.29; Hosmer–Lemeshow goodness‐of‐fit *p* = 0.42; overall classification accuracy = 71.5%; *n* = 400 (167 hypertension events). Diabetes status, age, sex, BMI, education, marital status, occupation, income, and smoking were the covariates retained in the final model per the a priori variable‐selection criteria described in Section 2.7.

Abbreviations: BMI, body mass index; CI, confidence interval; OR, odds ratio.

The final model was statistically significant, showed adequate fit (Hosmer–Lemeshow *p* = 0.42), explained 29% of the variance in hypertension status (Nagelkerke *R* ^2^ = 0.29), and correctly classified 71.5% of participants.

Health‐related quality of life, measured using the EQ‐5D‐5L instrument, differed significantly between participants with and without self‐reported diabetes (Table 4). The mean EQ‐5D‐5L index score was substantially lower among diabetic participants (0.72 ± 0.18) than among non‐diabetic participants (0.85 ± 0.12; *p* < 0.001).

**Table 4:** Comparison of EQ‐5D‐5L dimensions and index scores between diabetic and non‐diabetic participants.

EQ‐5D Dimension/Level	Diabetic, *N* = 200, *n* (%)	Non‐diabetic, *N* = 200, *n* (%)	*p*
1. Mobility problems			0.02
No problems	120 (60.0)	160 (80.0)	
Some problems	70 (35.0)	38 (19.0)	
Extreme problems	10 (5.0)	2 (1.0)	
2. Self‐care difficulties			0.14
No problems	165 (82.5)	165 (82.5)	
Some problems	32 (16.0)	30 (15.0)	
Extreme problems	3 (1.5)	5 (2.5)	
3. Problems with usual activities			0.07
No problems	105 (52.5)	170 (85.0)	
Some problems	80 (40.0)	20 (10.0)	
Extreme problems	15 (7.5)	10 (5.0)	
4. Pain/discomfort			0.02
No problems	90 (45.0)	130 (65.0)	
Some problems	95 (47.5)	65 (32.5)	
Extreme problems	15 (7.5)	5 (2.5)	
5. Anxiety/Depression			0.004
No problems	110 (55.0)	150 (75.0)	
Some problems	80 (40.0)	45 (22.5)	
Extreme problems	10 (5.0)	5 (2.5)	
6. EQ‐5D‐5L index score, mean ± SD	0.72 ± 0.18	0.85 ± 0.12	< 0.001

In the mobility domain, 120 of 200 diabetic participants (60%) reported no problems compared with 160 of 200 non‐diabetic participants (80%), while 70 (35%) of diabetics reported some problems versus 38 (19%) of non‐diabetics, and 10 (5%) of diabetics reported extreme problems versus 2 (1%) of non‐diabetics (*p* = 0.02).

For self‐care difficulties, no statistically significant difference was observed between groups (*p* = 0.14): 165 (82.5%) of diabetic and 165 (82.5%) of non‐diabetic participants reported no problems, with the remainder distributed similarly between the some‐problems and extreme‐problems categories.

Regarding usual activities, the between‐group difference approached but did not reach statistical significance (*p* = 0.07): 105 (52.5%) of diabetic participants reported no problems compared with 170 (85%) of non‐diabetic participants.

In the pain/discomfort domain, diabetic participants reported significantly greater burden (*p* = 0.02): only 90 (45%) reported no pain compared with 130 (65%) of non‐diabetics.

In the anxiety/depression domain, diabetic participants also reported significantly poorer outcomes (*p* = 0.004): no anxiety/depression was reported by 110 (55%) of diabetics compared with 150 (75%) of non‐diabetics.

To identify independent correlates of HRQoL, a multivariable linear regression model was fitted with the EQ‐5D‐5L index score as the continuous outcome (Table 5). After adjustment, diabetes status remained significantly and independently associated with lower HRQoL: diabetic participants scored, on average, 0.081 points lower on the EQ‐5D‐5L index than non‐diabetic participants (*β* = −0.081, 95% CI − 0.104 to −0.058, *p* < 0.001), holding other covariates constant.

**Table 5:** Multivariable linear regression analysis of factors associated with the EQ‐5D‐5L index score.

Characteristic	Unadjusted *β* (95% CI)	Adjusted *β* (95% CI)	*p*
Diabetes status			
No (Ref)	0.00	0.00	—
Yes	−0.130 (− 0.156 to −0.104)	−0.081 (− 0.104 to −0.058)	< 0.001
Age group			
45–54 (Ref)	0.00	0.00	—
55–64	−0.041 (− 0.068 to −0.014)	−0.028 (− 0.052 to −0.004)	0.02
≥ 65	−0.066 (− 0.093 to −0.039)	−0.047 (− 0.072 to −0.022)	< 0.001
BMI status			
Normal weight (Ref)	0.00	0.00	—
Overweight	−0.026 (− 0.050 to −0.002)	−0.019 (− 0.041 to 0.003)	0.09
Obese	−0.048 (− 0.076 to −0.020)	−0.035 (− 0.061 to −0.009)	0.008
Education			
No formal education (Ref)	0.00	0.00	—
Secondary & above	0.030 (0.002 to 0.058)	0.022 (− 0.004 to 0.048)	0.10
Income group			
High income (Ref)	0.00	0.00	—
Low income	−0.041 (− 0.070 to −0.012)	−0.031 (− 0.058 to −0.004)	0.02
Occupation			
Manual labour (Ref)	0.00	0.00	—
Non‐manual	−0.017 (− 0.040 to 0.006)	−0.011 (− 0.032 to 0.010)	0.30
Smoking status			
No (Ref)	0.00	0.00	—
Yes	−0.033 (− 0.060 to −0.006)	−0.024 (− 0.049 to 0.001)	0.06

*Note:* Model statistics: *R* ^2^ = 0.24; adjusted *R* ^2^ = 0.22; overall model *p* < 0.001; *n* = 400. Education and occupation categories with very small cell counts were collapsed as noted; see Table 1 for full category‐level frequencies.

Abbreviations: *β*, unstandardized regression coefficient; BMI, body mass index; CI, confidence interval.

Older age was independently associated with lower HRQoL: compared with participants aged 45–54 years, those aged 55–64 years scored 0.028 points lower (95% CI −0.052 to −0.004, *p* = 0.02), and those aged ≥ 65 years scored 0.047 points lower (95% CI − 0.072 to −0.022, *p* < 0.001). Obesity was independently associated with lower HRQoL (*β* = −0.035, 95% CI −0.061 to −0.009, *p* = 0.008), while the overweight category showed a similar but non‐significant trend (*β* = −0.019, 95% CI −0.041 to 0.003, *p* = 0.09). Low income was independently associated with lower HRQoL (*β* = −0.031, 95% CI −0.058 to −0.004, *p* = 0.02). Education, occupation, and smoking were not independently associated with the EQ‐5D‐5L index score after adjustment (Table 5).

The overall model was statistically significant (*F*‐test, *p* < 0.001) and explained 24% of the variance in EQ‐5D‐5L index scores (*R* ^2^ = 0.24, adjusted *R* ^2^ = 0.22).

Site‐level hypertension prevalence, examined descriptively, ranged from 37.5% to 47.5% across the five study slums (Table 6; overall combined prevalence 41.8%), with Mirpur showing the highest prevalence (47.5%) and Khilgaon and Kamalapur the lowest (37.5% each). Because per‐site samples were not powered for formal statistical comparison (Section 2.7), these estimates should be interpreted as descriptive and hypothesis‐generating rather than confirmatory.

**Table 6:** Site‐specific prevalence of self‐reported hypertension across the five study slums (exploratory, descriptive analysis).

Study slum	*n*	Hypertension, *n* (%)	95% CI
Mirpur	80	38 (47.5)	36.4–58.9
Khilgaon	80	30 (37.5)	27.0–49.1
Korail	80	36 (45.0)	34.0–56.5
Bhashantek	80	33 (41.3)	30.5–52.9
Kamalapur	80	30 (37.5)	27.0–49.1
Total	400	167 (41.8)	36.9–46.7

*Note:* Descriptive, exploratory analysis; per‐site samples were not powered for formal between‐site statistical comparison (Section 2.7). CI = confidence interval (Wilson score method).

Among the 200 participants with diabetes, financial constraints were the most commonly reported barrier to accessing care, cited by 167 participants (83.5%), followed by limited access to healthcare services (19 participants, 9.5%), lack of awareness (9 participants, 4.5%), and cultural factors (5 participants, 2.5%).

## 4 Discussion

This community‐based cross‐sectional study among 400 adults aged ≥ 45 years in five Dhaka urban slums revealed a substantial burden of hypertension among those with self‐reported diabetes (60.5%) compared with non‐diabetics (23.0%; Table 2), with site‐level hypertension prevalence ranging from 37.5% to 47.5% across the five slums (Table 6). This overall burden exceeds the 28.3% hypertension prevalence reported among adults ≥ 35 years in an earlier urban‐slum study in Bangladesh [15], likely reflecting our study's restriction to an older (≥ 45 years), and therefore higher‐risk, age group, together with the enrichment of our sample with a diabetic subgroup at elevated cardiometabolic risk. The elevated comorbidity between diabetes and hypertension observed here underscores the synergistic risk conferred by these two conditions in resource‐constrained urban‐poor settings, consistent with national trends showing rising NCD comorbidity that exceeds rural estimates [20, 21]. As detailed in the Introduction, diabetes was analyzed as the primary exposure and hypertension as the outcome in this study given the established pathophysiological pathways linking diabetes to secondary hypertension and the greater feasibility of community‐based diabetes ascertainment; the converse relationship (diabetes risk among hypertensive individuals) remains an important avenue for future investigation.

In the adjusted model, diabetes status was the single strongest independent correlate of hypertension (aOR 3.82), followed by older age, higher BMI, smoking, and non‐manual occupation (Table 3). These findings corroborate extensive literature from Bangladesh and other LMICs, where diabetes, advancing age, overweight/obesity, tobacco use, and low physical activity are established drivers of hypertension [21]. The graded BMI association reflects metabolic and hemodynamic changes amplified by urban dietary shifts and inactivity [22]. Bivariate screening (Section 2.7) also examined additional lifestyle factors, including dietary patterns (fruit, vegetable, oily food, and added‐salt intake) and walking frequency; as none of these met the *p* < 0.20 threshold for inclusion in the final parsimonious model, they are not further discussed here, though they remain worthy of investigation in adequately powered future studies.

HRQoL, measured by EQ‐5D‐5L, was significantly lower among diabetics, with greater impairments in mobility, pain/discomfort, and anxiety/depression (Table 4). These bivariate differences were confirmed in multivariable analysis, in which diabetes remained independently associated with a clinically meaningful decrement in EQ‐5D‐5L index score after adjustment for demographic and clinical confounders (*β* = −0.081 and Table 5), alongside independent contributions of older age, obesity, and low income. These domain‐specific burdens mirror patterns reported among Bangladeshi type 2 diabetes patients, where EQ‐5D‐5L scores indicate “average” HRQoL overall but pronounced decrements in physical and mental domains linked to disease duration, comorbidities, and socioeconomic stressors [23, 24].

Beyond these statistical associations, several structural and social mechanisms specific to slum environments likely mediate the comorbidity of diabetes and hypertension and its impact on quality of life. Chronic psychosocial stress arising from insecure tenure, overcrowding, and daily economic precarity activates the hypothalamic–pituitary–adrenal axis and sympathetic nervous system, promoting both insulin resistance and sustained blood pressure elevation. Poor built environments in slums—characterized by narrow lanes, absence of safe public space for physical activity, and proximity to open drains and solid‐waste dumps—limit opportunities for exercise while increasing exposure to air and water pollutants associated with vascular dysfunction [25, 26]. Food insecurity, somewhat paradoxically, is also implicated in NCD risk in this setting: irregular access to adequate nutrition promotes reliance on cheap, energy‐dense, low‐nutrient foods (refined carbohydrates, processed snacks, high‐sodium preserved items) that are disproportionately available and affordable in informal urban food markets, contributing to both dysglycemia and sodium‐related blood pressure elevation. These mechanisms plausibly operate synergistically rather than independently, meaning that interventions addressing only clinical risk factors (e.g., medication access) without addressing the underlying social and environmental determinants are unlikely to durably reduce the disease burden in this population [27].

Financial constraints dominated healthcare barriers among diabetic participants (167/200, 83.5%; Figure 1), far outweighing access (19/200, 9.5%), awareness (9/200, 4.5%), and cultural factors (5/200, 2.5%). This overwhelming economic barrier persists despite Bangladesh's nominally free public primary healthcare system, reflecting systemic failures at multiple levels: community clinics and union sub‐centers frequently experience stock‐outs of essential antihypertensive and antidiabetic medicines, forcing patients into out‐of‐pocket purchases from private pharmacies; routine monitoring tests (fasting glucose, HbA1c) are often unavailable at the primary‐care level and require costly referral to secondary facilities; and daily‐wage slum residents face substantial indirect costs from lost work time associated with clinic visits, which can outweigh direct medical costs. Under‐resourced primary facilities, chronic medicine shortages, and overburdened health workers further compound access issues, highlighting systemic gaps in NCD service integration at the primary healthcare level that existing infrastructure alone has not resolved [28, 29, 30].

Strengths of this study include its community‐based design in understudied slum settings, use of validated tools (WHO STEPS, EQ‐5D‐5L Bengali versions), and random sampling to enhance representativeness. Several limitations should be acknowledged. First, hypertension and, to a lesser extent, diabetes status were based on self‐report rather than direct clinical measurement; blood pressure was not measured at interview because of logistical constraints in the slum field setting (lack of calibrated equipment, cold chain, certified phlebotomists, and safe referral pathways for abnormal readings), and this is a key limitation of the descriptive hypertension estimates. Diabetes diagnosis was corroborated with documentary evidence for 78.0% of diabetic participants but relied on self‐report alone for the remainder. Second, recruitment was restricted to adults aged ≥ 45 years for the reasons outlined in Section 2.3; consequently, our findings do not capture younger‐onset diabetes or hypertension and should not be extrapolated to younger age groups. Third, the cross‐sectional design precludes causal inference. Fourth, purposive selection of the five study slums limits generalizability of findings to other slum or non‐slum settings. Fifth, the EQ‐5D‐5L is a generic instrument and may not capture all disease‐specific HRQoL concerns. Sixth, social desirability bias may have affected reporting of lifestyle behaviors. Seventh, the healthcare‐barriers analysis (Figure 1) was restricted to the diabetic subgroup (*n* = 200) and therefore does not describe barriers among non‐diabetic participants; this restriction, and its rationale, is detailed in Section 2.5. Eighth, although the events‐per‐variable ratio for the adjusted hypertension model (≈10) met conventional adequacy thresholds, the study was not originally powered for multivariable modeling, and adjusted estimates—particularly for less common exposure categories—should be interpreted with appropriate caution. Finally, despite random sampling within slums, residual selection and recall bias cannot be completely excluded.

## 5 Conclusion

This study highlights the considerable burden of diabetes and hypertension among adults living in the urban slums of Dhaka, Bangladesh, and underscores the complex health challenges faced by disadvantaged urban populations. The coexistence of chronic diseases, poorer health‐related quality of life, and difficulties in accessing healthcare reflects the broader vulnerability of slum communities to the consequences of non‐communicable diseases.

The findings suggest that the growing NCD burden in urban poor settings cannot be addressed through clinical care alone. Social and economic disadvantage, limited access to affordable healthcare, unhealthy living conditions, and barriers to adopting healthy lifestyles may collectively influence disease prevention, treatment, and long‐term outcomes. Therefore, reducing the impact of NCDs requires greater attention to the wider social and structural determinants of health alongside individual‐level risk factors.

A comprehensive and equity‐oriented approach is needed to strengthen NCD prevention, early detection, treatment, and long‐term management within underserved urban communities. Expanding community‐based health services, improving access to affordable medicines and diagnostics, strengthening primary healthcare, and promoting health literacy and healthy lifestyles could help reduce the burden of chronic disease. Greater integration of NCD services into existing community and primary healthcare systems may also improve continuity of care and reach populations that remain underserved.

Ultimately, addressing diabetes and hypertension in urban slums requires sustained investment, community engagement, and policies that recognize the socioeconomic realities of vulnerable populations. Such efforts are essential not only for reducing preventable illness and premature mortality but also for improving quality of life, protecting households from financial hardship, and reducing persistent health inequities in Bangladesh.

## Author Contributions

**Md Fakhrul Islam Maruf:** conceptualization, investigation, writing – original draft, methodology, validation, writing – review and editing, formal analysis, data curation, project administration, resources. **Nishat Tamanna Omi:** validation, writing – review and editing, writing – original draft, investigation, resources, data curation. **Mahfuza Mubarak:** supervision, investigation, writing – review and editing, validation.

## Funding

The authors have nothing to report.

## Disclosure

All authors have read and approved the final version of the manuscript. Md. Fakhrul Islam Maruf had full access to all of the data in this study and takes complete responsibility for the integrity of the data and the accuracy of the data analysis.

## Ethics Statement

Ethical approval was obtained from the Institutional Review Board of Jahangirnagar University, Savar, Dhaka, Bangladesh (Ref No: BBEC, JU/M 2025/09 (314)). The study was conducted between June and August 2025. Written informed consent was obtained from all participants prior to data collection. Participation was voluntary, and confidentiality of all information was strictly maintained.

## Consent

Verbal informed consent was taken from all participants before the interview. After the partcipants were informed and they provided verbal consent, they were asked questiones related to our study.

## Conflicts of Interest

The authors declare that they have no competing interests.

## Use of AI‐Assisted Tools

This manuscript was prepared by the listed human authors, who take full responsibility for its content. Large Language Models (LLMs), specifically ChatGPT‐5 (OpenAI), were used solely to assist in improving the clarity, grammar, and flow of the language. The authors reviewed and edited all AI‐generated text to ensure accuracy, integrity, and adherence to scientific standards. No content, data interpretation, or conclusions were generated autonomously by the AI tool.

## Transparency Statement

Md. Fakhrul Islam Maruf affirms that this manuscript is an honest, accurate, and transparent account of the study being reported; that no important aspects of the study have been omitted; and that any discrepancies from the study as planned have been explained.

## Data Availability

The data are available from the corresponding author (Md. Fakhrul Islam Maruf) upon reasonable request, subject to approval by the Institutional Review Board of Jahangirnagar University and compliance with data protection regulations. Requests should include a clear description of the intended use and may require a data‐sharing agreement to ensure participant confidentiality and appropriate use. The study questionnaire (adapted from the Bengali WHO STEPS tool) and the EQ‐5D‐5L instrument (validated Bengali version) are standardized tools. The WHO STEPS questionnaire is freely available from the World Health Organization website. The EQ‐5D‐5L is copyrighted by the EuroQol Research Foundation; permission for use was obtained, and the instrument can be requested via the EuroQol website.
